# Proteomic Analysis of the Effect of Inorganic and Organic Chemicals on Silver Nanoparticles in Wheat

**DOI:** 10.3390/ijms20040825

**Published:** 2019-02-14

**Authors:** Hafiz Muhammad Jhanzab, Abdul Razzaq, Yamin Bibi, Farhat Yasmeen, Hisateru Yamaguchi, Keisuke Hitachi, Kunihiro Tsuchida, Setsuko Komatsu

**Affiliations:** 1 Faculty of Life and Environmental and Information Sciences, Fukui University of Technology, Fukui 910-8505, Japan; jhanzabmuhammad200@yahoo.com; 2 Department of Agronomy, PMAS-Arid Agriculture University, Rawalpindi 46300, Pakistan; arazzaq57@yahoo.com; 3 Department of Botany, PMAS-Arid Agriculture University, Rawalpindi 46300, Pakistan; dryaminbibi@uaar.edu.pk; 4 Department of Botany, Women University, Swabi 23340, Pakistan; fyasmeen@wus.edu.pk; 5 Institute for Comprehensive Medical Science, Fujita Health University, Toyoake 470-1192, Japan; hyama@fujita-hu.ac.jp (H.Y.); hkeisuke@fujita-hu.ac.jp (K.H.); tsuchida@fujita-hu.ac.jp (K.T.)

**Keywords:** proteomics, wheat, silver nanoparticles

## Abstract

Production and utilization of nanoparticles (NPs) are increasing due to their positive and stimulating effects on biological systems. Silver (Ag) NPs improve seed germination, photosynthetic efficiency, plant growth, and antimicrobial activities. In this study, the effects of chemo-blended Ag NPs on wheat were investigated using the gel-free/label-free proteomic technique. Morphological analysis revealed that chemo-blended Ag NPs resulted in the increase of shoot length, shoot fresh weight, root length, and root fresh weight. Proteomic analysis indicated that proteins related to photosynthesis and protein synthesis were increased, while glycolysis, signaling, and cell wall related proteins were decreased. Proteins related to redox and mitochondrial electron transport chain were also decreased. Glycolysis associated proteins such as glyceraldehyde-3-phosphate dehydrogenase increased as well as decreased, while phosphoenol pyruvate carboxylase was decreased. Antioxidant enzyme activities such as superoxide dismutase, catalase, and peroxidase were promoted in response to the chemo-blended Ag NPs. These results suggested that chemo-blended Ag NPs promoted plant growth and development through regulation of energy metabolism by suppression of glycolysis. Number of grains/spike, 100-grains weight, and yield of wheat were stimulated with chemo-blended Ag NPs. Morphological study of next generational wheat plants depicted normal growth, and no toxic effects were observed. Therefore, morphological, proteomic, yield, and next generation results revealed that chemo-blended Ag NPs may promote plant growth and development through alteration in plant metabolism.

## 1. Introduction

Advancement in nanotechnology has led to the production of nanoparticles (NPs), which are extensively used in diversifying a range of applications and products [1]. NPs are atomic or molecular aggregates characterized by their small size of less than 100 nm [2] and have larger surface areas that radically modify their physicochemical properties in comparison to the bulk material [3]. Exposure of NPs to plants resulted in cellular production of reactive oxygen species (ROS) leading to both positive and negative effects [4]. Activity of NPs depends upon size, composition, surface area, and nature of metal materials [5]. Over production and utilization of NPs have raised serious concern about their impacts on the ecosystem [6]. The size and concentration of NPs are responsible for their interaction with other materials and have diverse effects on plants [7]. Plants are the primary and essential components of the ecosystem with the capability to accumulate NPs. Therefore, interaction of NPs with plants and environment needs investigation.

Majority of the studies on NPs have discovered significant and astounding effects on biological systems. Silver (Ag) NPs with concentration of 25 ppm and 50 ppm increased plant growth and biochemical parameters in mustard [8]. Ag NPs with 100 nm reduced biomass and transpiration of *Cucurbita pepo* [9]; and increased carbohydrates and protein contents of *Bacopa monnieri* [10]. However, higher concentration such as 1000 ppm of Ag NPs resulted in an increase of superoxide dismutase (SOD) in *Solanum lycopersicum* [11]. 50 ppm of Ag NPs increased root nodulation in cowpea, while 75 ppm of Ag NPs improved shoot parameters in brassica. In maize, 40 ppm of Ag NPs increased root and shoot growth, while 60 ppm of Ag NPs promoted germination [12]. Ag NPs’ exposure to explants of sugarcane stimulated growth at 50 ppm and inhibited at 200 ppm [13]. Ag NPs modified the gene expressions that were involved in cellular events, including cell proliferation, metabolism, and hormone signaling [14]. It is necessary to investigate the response of Ag NPs on a molecular basis to understand the morpho-physiological modifications in plants.

In plants, nicotinic acid as an important organic chemical, improving growth and productivity [15]. Nicotinic acid increased plant growth, protein synthesis [16] and enzyme activities, such as ascorbate per oxidase, glutathione, and fumarase. Damages of oxidative stresses can be protected by the application of nicotinic acid through DNA methylation [17]. Potassium nitrate (KNO_3_), as an inorganic chemical, plays an important role in most plants’ biochemical and physiological processes, such as photosynthesis, enzyme activation, energy transfer, and stress resistance [18]. Increment in plant growth and rate of photosynthesis in response to KNO_3_ ultimately improved the yield of wheat, maize, and potato [19]. Potassium minimized the cadmium toxicity, improved yield, mineral elements, and antioxidant defense system of *Cicer arietinum* [20]. These findings conclude that exogenous application of organic and inorganic chemicals promoted the growth and yield parameters of plants; however, their molecular and metabolic mechanisms are still not clear.

Findings related to proteomic studies revealed that Ag NPs maintained cellular homeostasis by changing proteins involved in redox regulation and sulfur metabolism [21]. The application of Ag NPs decreased alcohol dehydrogenase and pyruvate decarboxylase, while increased amino acid related proteins and wax formation in soybean under flooding stress [22]. Proteins responsible for oxidative stress such as tolerance, calcium regulation, signaling, cell division, and apoptosis were identified in response to Ag NPs in *Eruca sativa* [23]. Several proteins related to primary metabolism and cell defense in roots and shoots of wheat were altered with treatment of Ag NPs [21]. Proteomic studies on the effects of Ag NPs have been reported; however, the effect of Ag NPs blended with organic and inorganic chemicals have not been reported earlier. To study the effects of blended NPs on wheat, morphological, proteomic, and enzymatic analyses were performed.

## 2. Results

### 2.1. Growth Response of Wheat to Ag NPs Mixed with Organic and Inorganic Chemicals

**Figure 1:**
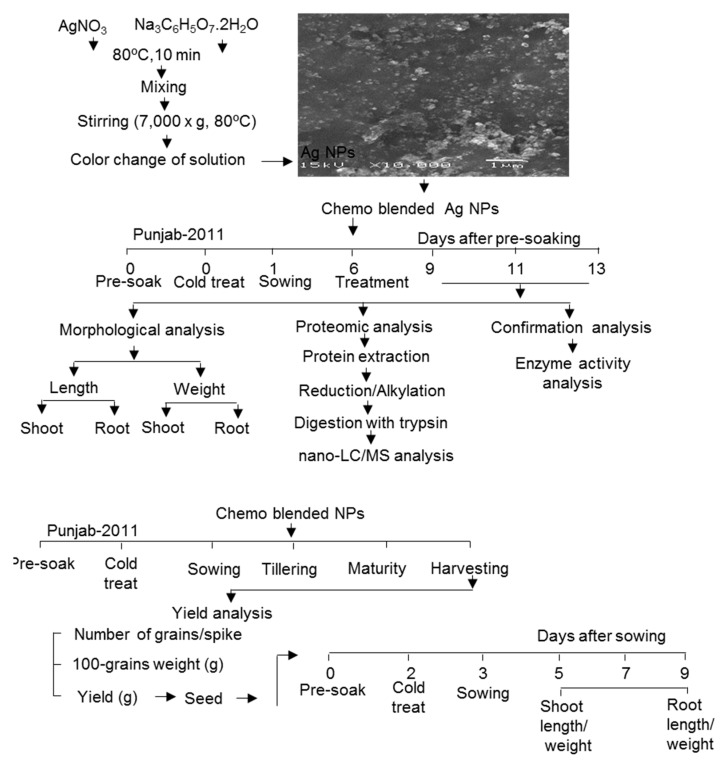
Experimental design for morphological, proteomics, confirmation analyses with subsequent impact on yield and next generation of wheat. Six-day-old wheat seedlings were treated with and without 5 ppm Ag NPs; 5 ppm Ag NPs/10 ppm nicotinic acid; 5 ppm Ag NPs/ 0.75% KNO_3_ and 5 ppm Ag NPs/10 ppm nicotinic acid/0.75% KNO_3_. Data regarding morphological parameters were analyzed on the 9th, 11th, and 13th day. For proteomic and confirmation analyses, proteins were extracted from shoot of 11-day-old treated plants and analyzed through gel free/label free proteomic technique. Thirty-two days old wheat plants were treated with chemo-blended Ag NPs. Number of grains/spike, 100-grains weight, and yield of wheat were analyzed. Seeds obtained from this lifecycle were used for next generation effect.

The experiment was conducted to evaluate the response of wheat to Ag NPs and Ag NPs mixed with organic and inorganic chemicals (Figure 1). The NPs were prepared by reduction of AgNO_3_ with Na_3_C_6_H_5_O_7_. 2H_2_O. SEM images revealed that Ag NPs are spherical with a size of 15–20 nm (Figure 1). Chemo-blended Ag NPs were prepared by mixing organic and inorganic chemicals. Wheat seeds were pre-soaked and followed by cold treatment for one day. Six-day-old wheat seedlings were treated with and without Ag NPs and chemo-blended Ag NPs. Data regarding morphological parameters were analyzed on the 9th, 11th, and 13th day (Figure 1). Proteins from treated as well as control plants were extracted and analyzed through gel free/label free proteomic technique. Antioxidant enzyme analysis was carried out to confirm the proteomic results. At the tillering stage, chemo-blended Ag NPs were applied and data on yield parameters were analyzed. Seeds obtained from this lifecycle were used to explore the cross-generational effects of nanoparticles. For this purpose, pre-sterilized seeds were sown and their growth attributes were analyzed on the 5th, 7th and 9th day (Figure 1).

**Figure 2:**
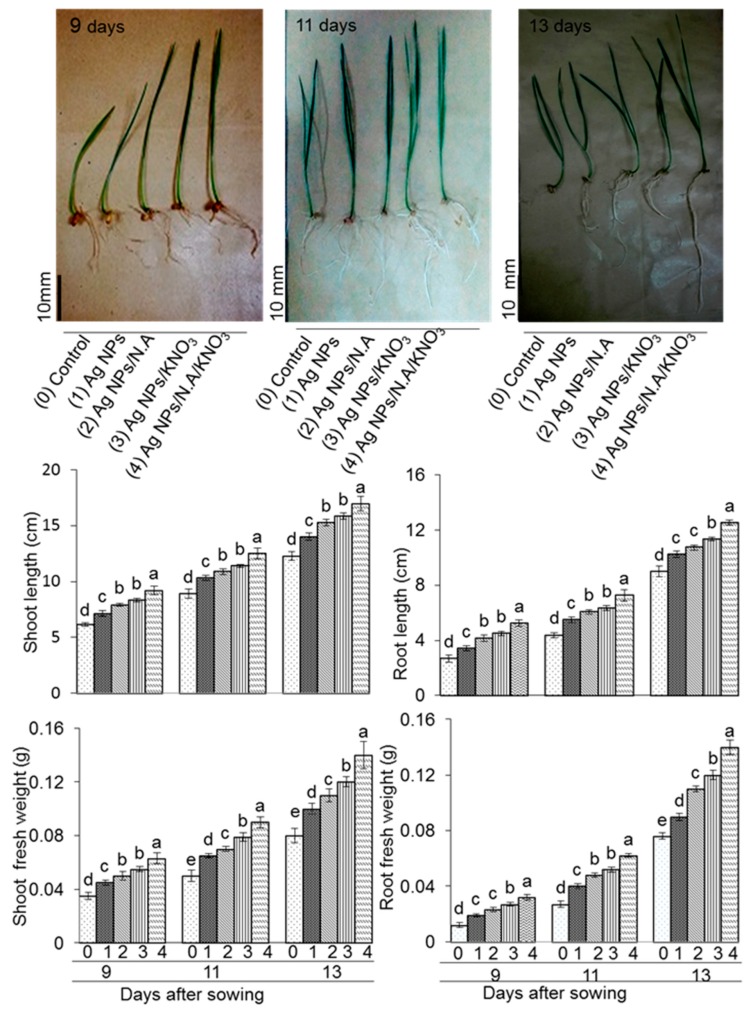
Morphological effects of chemo-blended Ag NPs on wheat. Six-day-old wheat seedlings were treated without and with 5 ppm Ag NPs, 5 ppm Ag NPs/10 ppm nicotinic acid, 5 ppm Ag NPs/0.75% KNO_3_, and 5 ppm Ag NPs/10 ppm nicotinic acid/0.75% KNO_3_. Photographs of wheat seedlings show the 9th, 11th, and 13th day with and without blended Ag NPs. Black bar in each photograph indicates size in mm. Shoot length, shoot fresh weight, root length, and root fresh weight were analyzed on the 9th, 11th, and 13th day. The data are presented as mean ± S.D. from three independent biological replicates. Mean values at each point with different letters (a,b,c,d) are significantly different according to Tukey’s multiple range test (p b 0.05).

Growth parameters such as shoot length, shoot fresh weight, root length, and root fresh weight were measured on the 9th, 11th, and 13th day after pre-soaking. Shoot length was significantly increased with 5 ppm Ag NPs/10 ppm nicotinic acid/0.75% KNO_3_ on the 9th, 11th, and 13th day compared to other treatments (Figure 2). Shoot fresh weight was maximum with 5 ppm Ag NPs/10 ppm nicotinic acid/0.75% KNO_3_ treated wheat plants on 9th, 11th, and 13th day measurements (Figure 2). Root length and root fresh weight were also greatly affected with application of Ag NPs mixed with organic and inorganic chemicals. Root length and root fresh weight were increased with 5 ppm Ag NPs/10 ppm nicotinic acid/0.75% KNO_3_ during the 9th, 11th, and 13th day (Figure 2). Comparative analysis indicated that length and weight of shoot and root were higher when chemo-blended Ag NPs was applied in comparison with control (Figure 2). Morphological parameters such as length and weight of shoot and root were affected by treatment of chemo-blended Ag NPs. Therefore, 5 ppm Ag NPs/10 ppm nicotinic acid/0.75% KNO_3_ was used for proteomic analysis.

### 2.2. Proteomic Analysis

Proteomic analysis was performed for identification of proteins that were affected in response to the chemo-blended Ag NPs. Protein extraction was carried out on 11-day-old treated wheat plants and analyzed through gel free/label free proteomic technique (Figure 1). The abundance of 49 proteins was significantly changed when treated with Ag NPs mixed with organic and inorganic chemicals (*p* < 0.05, Student’s *t* test). Out of these 49 proteins, 29 proteins increased (Table 1), while 20 proteins decreased on Ag NPs exposure (Table 2). According to GO term analysis, 49 proteins related to biological, cellular, and molecular processes were changed (Supplementary Figure S1). Analysis of GO term revealed that proteins related to the metabolism in biological processes were decreased/ increased on chemo-blended Ag NPs exposure, while in case of cellular processes, membrane related proteins were decreased/ increased on chemo-blended Ag NPs treatment (Supplementary Figure S1). For molecular functions, proteins related to catalytic activity were changed with abundance (Supplementary Figure S1).

**Figure 3:**
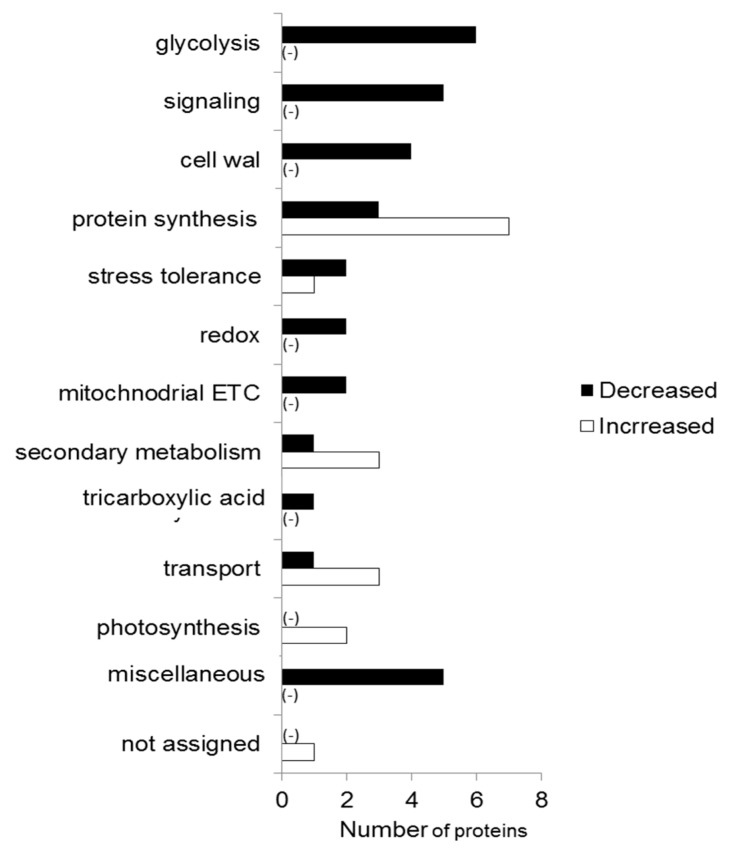
To determine the effect of Ag NPs mixed with organic and inorganic chemicals, MapMan software and KEGG database were used (Supplementary Figure S2). The proteins significantly changed were visualized through MapMan software (Supplementary Figure S2). Proteins related to cell wall, secondary metabolism, amino acid metabolism, photosynthesis, glycolysis, starch/sucrose synthesis, and lipid metabolism were significantly changed (Supplementary Figure S2).

The changed proteins were functionally categorized through MapMan bin code analysis (Figure 3). Proteins related to photosynthesis and protein synthesis were increased, while signaling, cell wall, and stress related proteins were decreased. The majority of proteins related to glycolysis were decreased in response to chemo-blended Ag NPs (Figure 3).

**Figure 4:**
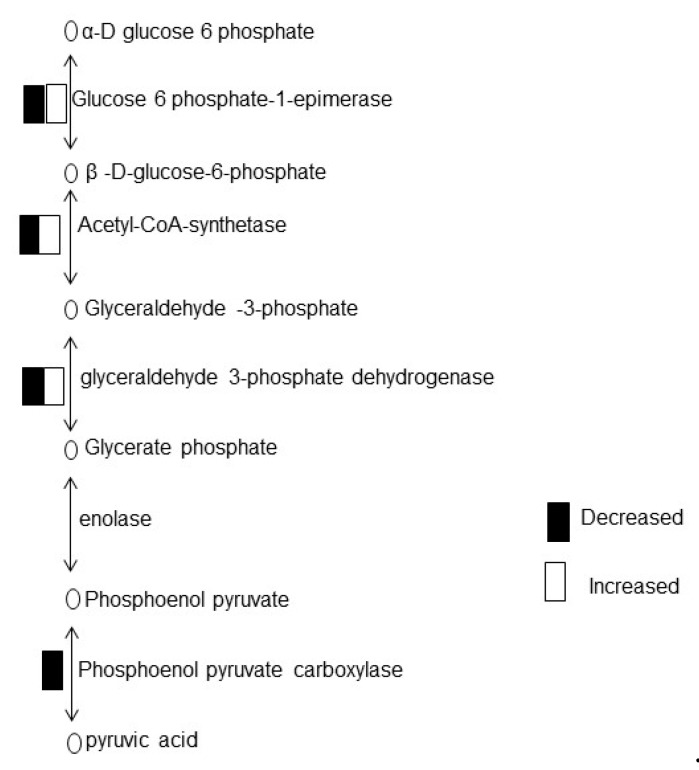
KEGG analysis of identified proteins on chemo-blended Ag NPs’ exposure. Pathway of glycolysis mapped by KEGG based on identified proteins treated with chemo-blended Ag NPs. Wheat plants were treated with Ag NPs mixed with organic and inorganic chemicals. Each square of black and white indicates a decreased or increased response, respectively, in ratio compared with untreated plants.

Proteins related to photosynthesis, protein synthesis, secondary metabolism, and transport were increased; while glycolysis, cell wall, and signaling-related proteins were decreased with treatment of chemo-blended Ag NPs. Further analysis was carried out to check the effect of chemo-blended Ag NPs on glycolysis. The identified proteins associated with glycolysis were mapped through a KEGG database (Figure 4). Among the glycolysis-related proteins, glyceraldehyde-3-phosphate dehydrogenase and glucose-6-phosphate-1-epimarase were increased/decreased after the chemo-blended Ag NPs treatment (Figure 4).

### 2.3. Effect of Chemo-Blended Ag NPs on Antioxidant Enzyme Activity Analysis of Wheat

**Figure 5:**
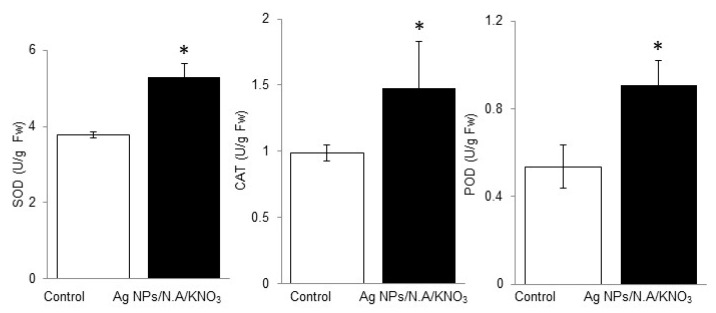
Effect of chemo-blended Ag NPs on the antioxidant activity of wheat. Wheat plants were treated with and without chemo-blended Ag NPs for six days. To assay the enzymatic activities, SOD, CAT and POD were extracted from leaves of wheat. Absorbance was recorded with a spectrophotometer. The data are presented ± S.D. from three independent biological replicates. The student *t*-test was used for statistical analysis. Significance between control and treated plants was indicated by asterisks (*P* < 0.05).

To confirm the proteomic results, antioxidant enzyme activities were analyzed. Antioxidant enzymes such as superoxide dismutase (SOD), catalase (CAT), and peroxidase (POD) were measured from fresh leaves of 11-day-old plants with and without chemo-blended Ag NPs. The results indicated that SOD activity was significantly increased with chemo-blended Ag NPs as compared to the control (Figure 5). CAT activity was also improved significantly on exposure to chemo-blended Ag NPs, as compared to the control. CAT as an important antioxidant enzyme is involved in conversion of H_2_O_2_ to water and oxygen, as well as scavenging of free radicals. (Figure 5). Similarly, POD activity was increased two times with treatment of Ag NPs mixed with organic and inorganic chemicals (Figure 5).

### 2.4. Effect of Chemo-Blended Ag NPs on Yield and Lifecycle of Wheat

**Figure 6:**
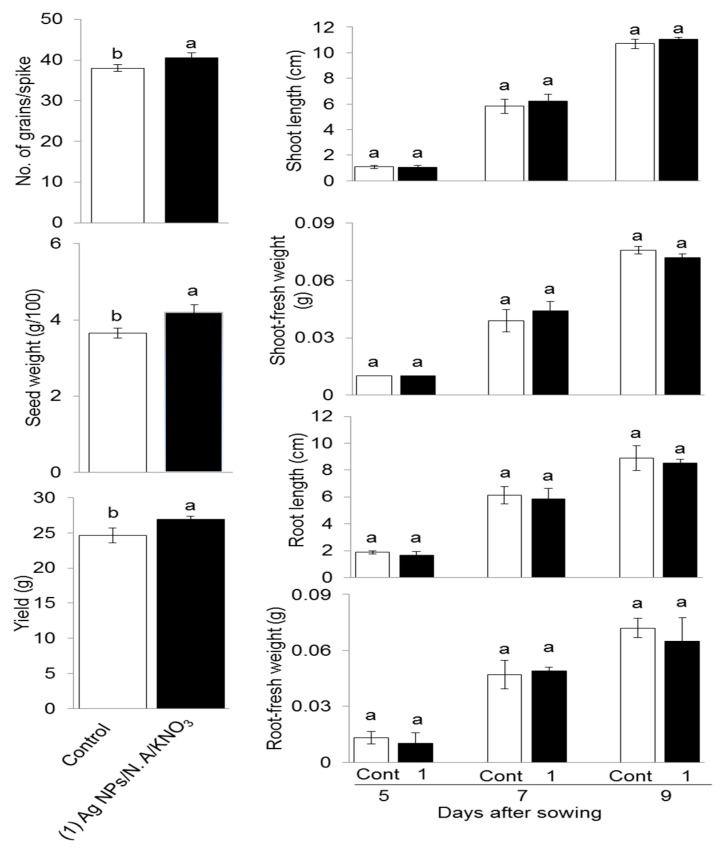
Effect of chemo-blended Ag NPs on yield and subsequent morphological assessment of the next generation of wheat seeds. Thirty-two day old wheat plants were treated without or with 5 ppm Ag NPs/10 ppm nicotinic acid/0.75% KNO_3_. Grains were harvested and number of grains/spike, 100-grains weight, and yield were analyzed. Seeds obtained from this life cycle were sown in sterilized sand. Shoot length, shoot-fresh weight, root length, and root-fresh weight were analyzed on the 5th, 7th, and 9th day after sowing. The data are presented as mean ± S.D. from three independent biological replicates. Mean value in each point with different letters are significantly different according to Tukey’s multiple range test (*P* < 0.05).

Exploring the possible effects of Ag NPs blended with organic and inorganic chemicals on the wheat yield, plants were treated without and with 5 ppm Ag NPs/10 ppm N.A/0.75% KNO_3_ at the tillering stage and allowed to grow till maturity. The grains were harvested at maturity and number of grains/spike, 100-grains weight, and yield was analyzed. Application of 5 ppm Ag NPs/10 ppm N.A/0.75% KNO_3_ promoted number of grains/spike, 100-grains weight and yield when compared to the control (Figure 6). Ag NPs blended with organic and inorganic chemicals promoted the production of crop and increased net grain yield.

Seeds obtained from this life cycle were used to analyze the transfer of chemo-blended Ag NPs impact on the next generation through a morphological experiment. After sterilization, pre-soaking and cold treatment, the seeds were sown in sterilized silica sand. Shoot length, shoot fresh weight, root length, and root fresh weight were analyzed on the 5th, 7th, and 9th day after sowing. Shoot length was higher with 5 ppm Ag NPs/10 ppm N.A/0.75% KNO_3_ as compared to control at the 7th and 9th day (Figure 6). Shoot fresh weight was maximum on the 7th day and reduced on the 9th day with 5 ppm Ag NPs/10 ppm N.A/ 0.75% KNO_3_. Root length was maximum with control, while root fresh weight was maximum on the 7th day with 5 ppm Ag NPs/ 10 ppm N.A/ 0.75% KNO_3_ as compared to the control (Figure 6). The results revealed no change in the morphology of the next generation of wheat plants. These results indicated that the application of chemo-blended Ag NPs did not show any inhibitory effect on the next generation. Normal and healthy plant growth was observed during the experiment.

## 3. Discussion

### 3.1. Effect of Chemo-Blended Ag NPs on Morphological Attributes of Wheat

To elucidate the effects of Ag NPs blended with organic and inorganic chemicals on wheat growth, wheat was treated with and without Ag NPs and blended Ag NPs. Wheat growth parameters were promoted in response to different treatments as compared to the control. Phyto-stimulatory as well as detrimental effects have been reported in several studies depending upon size and concentration of NPs. Ag NPs increased the super oxide dismutase activity in *Solanum lycopersicum* [11], while various concentrations of Ag NPs reduced plant biomass and transpiration [9]. Priming rice seeds with 5 ppm and 10 ppm of Ag NPs significantly increased germination, seedling vigor, and alpha amylase activity, resulting in higher soluble sugar contents [24]. Growth and photosynthesis were inhibited with 1 mM and 3 mM of Ag NPs and AgNO_3_, respectively [25]. Application of Ag NPs resulted in the increase in germination and chlorophyll contents of rice, maize and, peanut [26]. Low concentration such as 40 ppm of Ag NPs increased in vitro growth of root and shoot growth of maize, while 60 ppm concentration promoted the germination [12]. The present and earlier studies showed that NPs can stimulate the growth of various crops when applied in diverse concentrations.

Ag NPs with concentration of 1 mg/kg in soil did not affect growth and amino acid contents in wheat [27], while pea seeds treated with Ag NPs significantly promoted root length [28]. Coated Ag NPs with CTAB increased uptake of Ag in the roots, while reducing root growth and oxidative damages [7]. Ag NPs with 1000 µM and 3000 µM decreased growth, photosynthetic pigments, and chlorophyll fluorescence due to increased accumulation of Ag in root and shoot of pea seedlings [29]. Ag NPs reduced shoot length in mung bean [19] and barley [30], while germination of lentil was increased [31]. In the present study, Ag NPs mixed with organic and inorganic chemicals promoted growth of wheat. Individual effects of Ag NPs have been reported; however, Ag NPs mixed with organic and inorganic chemicals have not been reported earlier. These results revealed that Ag NPs can promote plant growth; therefore, fate and translocation of NPs to food chain needed more exploration.

### 3.2. Chemo-Blended Ag NPs Affect Protein Metabolism of Wheat

Proteomic analysis revealed that proteins related to protein synthesis were increased in response to Ag NPs mixed with organic and inorganic chemicals. Ag NPs increased proteins related to amino acid metabolism compared to flooding stress in soybean [32]. Ribosomal proteins have very crucial role in cell metabolism to regulate plant growth [33]. In stress conditions ribosomal proteins decreased [34] but Al_2_O_3_ NPs increased abundance of proteins related to protein synthesis [35]. Iron NPs increased photosynthesis and protein metabolism related proteins [36], while Ag NPs increased carbohydrates and protein contents of *Bacopamonnierei* [10]. Treatment of Ag NPs caused variation of proteins associated to endoplasmic reticulum and vacuole indicating the target organelles of Ag NPs [37]. These results indicated that chemo-blended Ag NPs increased protein synthesis that lead to increased growth and development of plants.

### 3.3. Chemo-Blended Ag NPs Affect Glycolysis of Wheat

Proteomic analysis showed that glycolysis related proteins were decreased in response to chemo-blended Ag NPs. Glycolysis is an important metabolic pathway responsible for conversion of glucose to pyruvate for production of energy [38]. In this process, energy is released from high energy molecules to regulate normal functions of plant growth and development. Plants change carbohydrate metabolic pathways to support ATP production through glycolysis [39]. In glycolysis, glyceraldehyde dehydrogenase converts glucose to energy and carbon molecules for metabolism of glycogen [40]. Proteins related to glycolysis were decreased in soybean upon exposure to Ag NPs [32], while increased with iron and copper NPs [41]. Taken together, these results suggest that glycolysis related proteins regulate energy metabolism in wheat through glyceraldehyde -3-phosphate dehydrogenase and phosphoenol pyruvate carboxylase.

### 3.4. Impact of Chemo-Blended Ag NPs on Scavenging Activity of SOD, CAT, and POD

Reactive oxygen species (ROS) are an intricate part of normal cellular physiology. ROS inhibit multiple glycolytic enzymes, including glyceraldehyde-3-phosphate dehydrogenase, pyruvate kinase M2, and phosphofructokinase-1. Consistently, glycolytic inhibition promotes flux into the oxidative arm of the pentose phosphate pathway to generate NADPH [42]. Redox homeostasis in plants is maintained through antioxidant machinery such as SOD, CAT, POD, and some low molecular non-enzymatic compounds like phenolics, flavonoids, terpenoids, tocopherols, and carotenoids [43]. SOD, CAT, and POD are important antioxidant enzymes having indispensable role in ROS detoxification. Generally, antioxidant enzymes such as SOD, CAT, and POD alter in response to change in ROS concentration [44]. SOD converts O_2_^−^ radicals to H_2_O_2_, and then H_2_O_2_ reduces to water and oxygen by CAT and POD. Therefore, it is the first line of defense system which prevents the cell from further injuries [45].

Exposure of NPs to plants resulted in cellular production of ROS [4]. Ag NPs increased SOD, CAT, and POD activities in *Spirodela polyoriza* [46], while activities of these enzymes remain same with high concentration of Al_2_O_3_ NPs [47]. Application of Ag NPs increased SOD, CAT, and POD activities in water hyacinth, while CAT and POD reduced production of ROS in *Bacopa monirei* [10]. Exposure of Ag NPs increased SOD, CAT, and POD in water hyacinth plant roots [48], while level of lipid per oxidation was reduced through increased activity of SOD, CAT, and POD in *Phanerochaete chryosporium* [49]. Ag NPs mixed with organic and inorganic chemicals increased SOD, CAT, and POD activities. In our study, glycolysis related proteins were decreased; however, activities of these proteins were increased due to stimulation of enzyme activities.

### 3.5. Effect of Chemo-Blended Ag NPs on Yield and Growth of Next Generation

To study the possible role of blended Ag NPs on yield and yield components of wheat, wheat plants were treated with Ag NPs mixed with organic and inorganic chemicals. Wheat yield was promoted with blended Ag NPs. In another study, it has been reported that Ag NPs of low doses 25–50 mg kg^−1^ increased chlorophyll contents, nitrate reductase, and *Phaselous vulgaris* pod yield [50]. Ag NPs increased number of grains/spike, 100-grains weight, and yield of wheat, while exposure of Fe and Cu NPs promoted yield and yield attributes of wheat [51]. Cesium NPs increased fruit production in tomato [52], while it reduced yield in cucumber [53]. Copper NPs increased number of grains/spike, 100-grains weight, and yield of wheat [54], while Ag NPs increased yield of cucumber [55]. Nano iron oxide increased pod dry weight and grain yield of soybean [56], while low concentration of Ag NPs did not affect growth and ascorbic acid contents of wheat seeds [27]. Ag NPs with magnetic field increased muskmelon fruit quality, yield, and soluble solid concentration [57]. Zinc oxide NPs coated with natural phytochemicals increased growth, biochemical parameters, and biomass of cotton significantly [58]. From these results, it can be concluded that application of NPs blended with organic and inorganic chemicals have great potential for increasing yield and yield attributes of plants.

In the present experiment, lifecycle study was carried out through application of Ag NPs blended with organic and inorganic chemicals. Seeds obtained from this life cycle were germinated and analyzed for growth responses. In a related study, treatment of radish plants with copper oxide and zinc oxide NPs through their life cycle indicated that root length, shoot length, and biomass in F1 seedlings were reduced [19]. A low concentration of cesium oxide NPs through their lifecycle on seed quality and next generation seedlings was obtained from treated parent plants with smaller biomass, reduced water transpiration and high ROS [52]. Multi-generational exposure of cesium oxide NPs to *Brassica rapa* showed slower plant growth and reduced biomass in the second and third generations. The number of seeds produced per siliqua was reduced in the third generation [47]. Pumpkin plants grown in an aqueous medium containing magnetite iron oxide NPs can absorb, translocate, and accumulate particles in plant tissues [59]. Ajirloo et al. [60] reported that a nanofertilizer of potassium and nitrogen increased growth, yield, and yield components of tomato. In the present study, plant growth was not affected; thus, chemo-blended Ag NPs act like a fertilizer, with no toxic effects on the next generation. Therefore, the current results provide future directions on cross generation studies related to NPs, in a bid to clearly understand the interaction of NPs and the environment.

## 4. Materials and Methods

### 4.1. Preparation of Chemo-Blended Nanoparticles

Ag NPs were synthesized by the reduction of silver nitrate (AgNO_3_) with trisodium citrate dihydrate (Na_3_C_6_H_5_O_7_. 2H_2_O). A solution of 500 ppm AgNO_3_ (Sigma-Aldrich, Munich, Germany) and 300 ppm Na_3_C_6_H_5_O_7_ (Merck, Darmstadt, Germany) were prepared. Prior to mixing of both solutions, AgNO_3_ solution was heated at 80 °C on a hot plate for 10 min. Trisodium citrate solution was gradually added to the AgNO_3_ solution and mixed thoroughly. The resultant solution was stirred at 7000× *g* for 1 h at 80 °C using a magnetic stirrer until a golden yellow color was attained [61]. Freshly prepared Ag NPs were analyzed through scanning electron microscopy image analysis.

Chemo-blended Ag NPs were prepared by mixing organic and inorganic chemicals. For the preparation of chemo-blended Ag NPs, Ag NPs, nicotinic acid (Sigma Aldrich, Darmstadt, Germany), and KNO_3_ (Sigma Aldrich, Darmstadt, Germany) were used. Different concentrations of 5 ppm Ag NPs, 10 ppm nicotinic acid, and 0.75% KNO_3_ were mixed in various combinations to prepare the blended NPs.

### 4.2. Plant Material and Treatment

Seeds of wheat (*Triticum aestivum* L. varPunjab-2011) were used to study the effects of Ag NPs mixed with organic and inorganic chemicals on the morphological and proteomic analysis of wheat. The seeds were sterilized with 2% sodium hypochlorite solution, followed by rinsing twice with water. After cold treatment at 4 °C, the seeds were sown in a plastic case containning pre-sterilized silica sand. Healthy and equal size seedlings were selected and placed on petridishes containing two layers of filter papers, covered by a sponge. Growth conditions were maintained as 16 h light intensity of 200 µmol m^−2^ s^−1^ and 8 h dark with 20% humidity at 25 °C. Six-day-old wheat seedlings were treated with and without 5 ppm Ag NPs, 5 ppm Ag NPs/10 ppm nicotinic acid, 5 ppm Ag NPs/0.75% KNO_3_ and 5 ppm Ag NPs/10 ppm nicotinic acid/0.75% KNO_3_. Shoot length, shoot-fresh weight, root length, and root-fresh weight were analyzed on the 9th, 11th, and 13th day after sowing. Three independent experiments were performed as biological replicates for all experiments. The plants used for biological replicates were sown on different days (Figure 1).

An experiment was conducted to evaluate the response of blended Ag NPs on yield of wheat. The seeds were sterilized and pre-soaked for two days. After cold treatment at 4 °C for one day, the seeds were sown in clay pots filled with fertile and thoroughly mixed soil. Thirty two-day-old plants were treated without and with blended nanoparticles, 5 ppm Ag NPs/10 ppm nicotinic acid/0.75% KNO_3_. Proper irrigation was managed during the critical stages of crop growth. Number of grains/spike, 100-grains weight and yield were analyzed after harvesting complete ripened grains from the spikes (Figure 1).

Seeds obtained from the above lifecycle were used for a morphological experiment. Sterilization was carried out with 2% NaOCl solution for 2 min followed by rinsing and pre-soaking for 2 days. After cold treatment at 4 °C for one day, the seeds were sown and allowed to grow in growth chamber at 25 °C, illuminated with white flourescent light of 200 µmol m^−2^ s^−1^ for 16-h light/day. Growth parametres such as shoot length, shoot-fresh weight, root length, and root-fresh weight were analyzed on the 5th, 7th, and 9th day after sowing. Three independent experiments were performed as biological replicates for all experiments. Sowing of seeds were carried out on different days to make biological replicates (Figure 1).

### 4.3. Protein Extraction

A portion (300 mg) of the sample was cut and ground for 60 times in a filter cartridge. It was ground for 30 times after adding 100 µL of lysis buffer containing 7 M urea, 2 M thiourea, 5% CHAPS, and 2 mM tributylphosphine. Furthermore, 50 µL of lysis buffer was added and ground for 30 times. The suspension was incubated for 2 min at 25 °C and centrifuged at 15,000× *g* for 2 min at 25 °C. Later on, the filter cartridge was removed and the supernatant was collected as total proteins.

### 4.4. Protein Enrichment, Reduction, Alkylation and Digestion

Extracted proteins (100 µg) were adjusted to a final volume of 100 µL. Methanol (400 µL) was added to each sample and mixed before addition of 100 µL of chloroform and 300 µL of water. After mixing and centrifugation at 20,000× *g* for 10 min to achieve phase separation, the upper phase was discarded and 300 µL of methanol was added to the lower phase, and then centrifuged at 20,000× *g* for 10 min. The pellet was collected as the soluble fraction [62]. The proteins were resuspended in 50 mM NH_4_HCO_3_, reduced with 50 mM dithiothreitol for 30 min at 56 °C, and alkylated with 50 mM iodoacetamide for 30 min at 37 °C in the dark. Alkylated proteins were digested with trypsin (Wako, Osaka, Japan) at a 1:100 enzyme/protein ratio for 16 h at 37 °C. Peptides were desalted with a MonoSpin C18 Column (GL Sciences, Tokyo, Japan). Peptides were acidified with 0.1% formic acid and analyzed by nano-liquid chromatography (LC) mass spectrometry (MS)/MS.

### 4.5. Measurement of Protein and Peptide Concentrations

The method proposed by Bradford [63] was used to determine the protein concentration with bovine serum albumin used as the standard. A Direct Detect Spectrometer (Millipore, Billerica, MA, USA) equipped with the Direct Detect software (version 3.0.25.0) was used to determine peptide concentration.

### 4.6. Protein Identification Using Nano LC-MS/MS

The samples were then analyzed using a LC system (EASY-nLC 1000; Thermo Fisher Scientific) coupled to a MS (Orbitrap Fusion ETD MS; Thermo Fisher Scientific). The LC conditions as well as MS acquisition conditions are described in the previous study [64]. Briefly, the peptides were loaded onto the LC system equipped with a trap column (Acclaim PepMap 100 C18 LC column, 3 µm, 75 µm ID × 20 mm; Thermo Fisher Scientific) equilibrated with 0.1% formic acid and eluted with a linear acetonitrile gradient (0–35%) in 0.1% formic acid at a flow rate of 300 nL/min. The eluted peptides were loaded and separated on the column (EASY-Spray C18 LC column, 3 µm, 75 µm ID × 150 mm; Thermo Fisher Scientific) with a spray voltage of 2 kV (Ion Transfer Tube temperature: 275 °C). The peptide ions were detected using the MS with the installed Xcalibur software (version 4.0; Thermo Fisher Scientific).

### 4.7. MS Data Analysis

The MS/MS searches were carried out using MASCOT (Version 2.6.1, Matrix Science, London, U.K.) and SEQUEST HT search algorithms against the UniProtKBTriticumaestivum database (2017-07-05) using Proteome Discoverer (PD) 2.2 (Version 2.2.0.388; Thermo Scientific). The search parameters were described previously [64].

### 4.8. Differential Analysis of Proteins Using MS Data

Label-free quantification was also performed with PD 2.2 and the differential analysis of the relative abundance of proteins between samples was performed using the PERSEUS software (version 1.6.0.7) [65], as previously described [64].

### 4.9. Functional Categorization

The protein sequences of the differentially changed proteins were subjected to a BLAST query against the Ami gene ontology (GO) database (http://amigo1.geneontology.org/cgi-bin/amigo/blast.cgi). The corresponding GO terms were extracted from the most homologous proteins using a Perl program. The GO annotation results were plotted by the Web Gene Ontology Annotation Plot (WEGO) (http://wego.genomics.org.cn/cgi-bin/wego/index.pl) tool by uploading compiled WEGO native format files containing the obtained GO terms. The gene functional annotations and protein categorization was analyzed using MapMan bin codes [66] and protein abundance ratio was assessed through MapMan software [67]. The MapMan software is generally linked with several external databases, which enable accurate measurement (http://mapman.gabipd.org). Pathway mapping of identified proteins was performed using Kyoto Encyclopedia of Genes and Genomes (KEGG) databases [68] (http://www.genome.jp/kegg/).

### 4.10. Analysis of Superoxide Dismutase, Catalase Activity, and Peroxidase in Response to Chemo-Blended Ag NPs

For analyses of change in enzyme activities on chemo-blended Ag NPs, fresh leaves (0.5 g) were ground in liquid nitrogen and homogenized in sodium phosphate buffer. The homogenate was centrifuged at 12,000× *g* for 15 min at 4 °C, and supernatant was collected in another tube. SOD activity was assessed by the method illustrated by Beauchamp and Fridovich [69] with little modifications. Catalase (CAT) activity was measured by the method described by Aebi and Bergmeyer [70] by analyzing decrease in H_2_O_2_ content at 240 nm with slight modifications. Peroxidase (POD) activity was analyzed using the guaiacol oxidation method by Li et al. [71].

### 4.11. Statistical Analysis

Data were analyzed by one-way ANOVA followed by Tukey’s multiple comparison among multiple groups using SPSS (version 22.0; IBM). A *p*-value of less than 0.05 was considered as statistically significant. Student *t*-test was used for comparison between two groups for statistical analysis. Significance (*P* < 0.05) among groups was indicated through asterisks.

## 5. Conclusions

Production and utilization of NPs are increasing in the eco-system. Plants as primary components of eco-system are more prone to accumulation of NPs, indicating the importance of the interaction of NPs with plants and the environment. The phytostimulatory effects of Ag NPs have been reported in several studies; however, the effects of chemo-blended Ag NPs have not been explored earlier. To investigate the mechanism of the effect of chemo-blended Ag NPs on wheat growth, a gel-free/label-free proteomic technique was used. The key findings of the current study are as follows: (i) The morphological analysis depicted that chemo-blended Ag NPs increased plant growth. (ii) Proteins related to secondary metabolism, protein synthesis, and transport were increased. (iii) Number of proteins related to glycolysis, signaling, and cell wall were decreased. (iv) Similarly, proteins related to redox and mitochondrial ETC also decreased. (v) In glycolysis, glyceraldehyde-3-phosphate dehydrogenase increased/decreased, while phosphoenol pyruvate carboxylase decreased. (vi) Enzymatic activities of SOD, POD, and CAT increased when chemo-blended AgNPs were tested on wheat. (vii) Chemo-blended Ag NPs promoted yield and yield components of wheat. (viii) Morphological analysis of the next generation showed normal growth without any toxic effects. Furthermore, maintenance of redox homeostasis through regulation of glycolysis and increased activities of antioxidant enzymes regulates energy metabolism. This maintenance of energy-related activities may stimulate plant growth and development in response to chemo-blended Ag NPs.

## Figures and Tables

**Table 1:** List of increased proteins identified on chemo-blended Ag NPs’ exposure in wheat.

No	Accession	Description	Difference	Functional Category	Biological Process	Cellular Component	Molecular Function
1	W5AYF4	Putative SNAP receptor protein	3.56	Transport	Cell organization and biogenesis	membrane	protein binding
2	A0A1D5WZM5	ER membrane protein complex	3.2	Protein	Transport	membrane	metal ion binding
3	A0A1D6B0Y2	At4g14100-like	2.91	Transport	Not assigned	membrane	catalytic activity
4	W5DZQ3	Ribosomal protein S1	2.85	Protein	Response to stimulus	chloroplast	RNA binding
5	Q5G1T9	Gamma-glutamylcysteinesynthetase	2.57	Protein	Metabolic process	Chloroplast, cytosol	catalytic activity
6	W5EA17	D-ribose 5-phosphate	2.41	secondary metabolism	Metabolic process	cytosol	catalytic activity
7	A0A1D5UQX6	Unknown	2.27	not assigned	Not assigned	not assigned	not assigned
8	W5DL10	Glutamate--tRNA ligase	1.96	Protein	Metabolic process	cytoplasm	catalytic activity
9	A0A1D5YQ15	Ferritin	1.86	photosynthesis	Cellular homeostasis	cytosol	catalytic activity
10	W5APX0	GrpE protein homolog	1.81	Transport	Metabolic process	mitochondrion	enzyme regulator activity
11	A0A1D6AQL7	PPIasecyclophilin-type	1.77	Stress	Metabolic process	cytosol	catalytic activity; protein binding
12	A0A1D5Y2E6	Reverse transcriptase	1.77	Protein	Metabolic process	Cytoplasm	catalytic activity
13	A0A1D5SUT9	Peptidase A1	1.76	Protein	Metabolic process	Membrane	catalytic activity
14	O21432	Ribosomal protein S2	1.65	Protein	Metabolic process	mitochondrion	structural molecule activity
15	A0A1D6AKZ2	ATP-dependent Clp protease proteolytic subunit	1.16	photosynthesis	Metabolic process	chloroplast	catalytic activity
16	A0A1D5V5A5	Bifunctional inhibitor/plant lipid transfer	1.12	secondary metabolism	Transport	Membrane	catalytic activity; metal ion binding
17	A0A1D5YXC6	Plant lipid transfer protein	1.01	secondary metabolism	Metabolic process	Membrane	catalytic activity
18	W5DX10	Allene oxide synthase-lipoxygenase	0.89	Stress	Metabolic process	chloroplast	catalytic activity; metal ion binding
19	A0A1D5YZ95	Synaptotagmin-like mitochondrial lipid-binding proteins	0.88	Transport	Metabolic process	Membrane	metal ion binding
20	A0A1D6DJK9	Tyrosine--tRNA ligase	0.86	Protein	Metabolic process	Cytoplasm	catalytic activity
21	A0A1D6RMY5	Short-chain dehydrogenase/reductase2	0.74	Stress	Metabolic process	cytosol	catalytic activity
22	W5QKZ0	Chalcone-flavonone isomerase	0.7	secondary Metabolism	Metabolic process	not assigned	catalytic activity
23	A0A1D5SIK2	NAD(P)H-quinone oxidoreductase subunit I,	0.7	ETC	metabolic process	membrane	catalytic activity; metal ion binding
24	A0A1D5S4W8	glyeraldehyde dehydrogenase	0.69	Glycolysis	metabolic process	membrane	catalytic activity
25	W5AV30	Acetyl-CoA synthetase	0.66	Glycolysis	not assigned	membrane	protein binding
26	Q06I94	Fasciclin-like protein FLA12	0.59	Cell wall	response to stimulus	membrane	not assigned
27	A0A1D5UQL1	Glucose-6-phosphate 1-epimerase	0.57	Glycolysis	metabolic process	chloroplast	catalytic activity
28	A0A1D6A8Y7]\	2-oxoglutarate (2OG) and Fe(II)-dependent oxygenase	0.53	Primary metabolism	metabolic process	cytoplasm	catalytic activity
29	A0A1D6RZJ3	N-acetyltransferase	0.4	Signaling	metabolic process	not assigned	catalytic activity

Ratio, relative abundance of protein; *p*-value b 0.05. Treated over control, wheat plant treated with Ag NPs compared with control.

**Table 2:** List of decreased proteins identified on chemo-blended Ag NPs’ exposure in wheat.

No	Accession	Description	Difference	Functional Category	Biological Process	Cellular Component	Molecular Function
1	A0A1D6C3J5	Copper transport protein	−0.65	Redox	Transport	membrane	metal ion binding
2	A0A1D6S991	Isocitrate dehydrogenase	−0.66	TCA	Metabolic process	Mitochondrion	catalytic activity
3	A0A1D5UMA5	3-Oxoacyl- synthase III	−0.71	lipid metabolism	Metabolic process	Chloroplast	catalytic activity
4	W5BFA5	glyceraldehyde-3-phosphate dehydrogenase	−0.72	Glycolysis	Metabolic process	Cytosol; Golgi	catalytic activity
5	A0A1D5V012	NADH dehydrogenase ubiquinone Fe-S protein 4	−0.74	Redox	Metabolic process	mitochondrion	catalytic activity
6	A0A1D5U7K0	F-box associated interaction domain	−0.89	Signaling	Regulation of biological process	cytoplasm	protein binding
7	A0A1D5VAF5	glycine-tyrosine-phenylalanine	−0.94	Stress	Metabolic process	mitochondrion	protein binding
8	A0A1D5XH81	carboxy peptidase	−0.97	Glycolysis	Metabolic process	chloroplast	catalytic activity
9	A0A1D6AAU8	acetyltransferase-superfamily	−1.11	Signaling	Metabolic process	not assigned	catalytic activity
10	A0A1D6AVB7	Ubiquitin-associated domain	−1.17	Protein	Response to stimulus	cytosol	protein binding
11	A0A1D6D1I9	Glutaredoxin	−1.18	ETC	Cellular homeostasis	mitochondrion	catalytic activity
12	A0A1D6AQL9	Rho termination factor, N-terminal domain superfamily	−1.26	primary metabolism	Metabolic process	mitochondrion	catalytic activity
13	A0A1D5ZVW9	La-type RNA-binding	−1.63	RNA	Metabolic process	cytosol	RNA binding
14	A0A096ULK3	Small GTPase superfamily	−1.76	Signaling	Regulation of biological process	cytosol	catalytic activity
15	A0A1D6RV75	peptidyl-prolyl cis-trans isomerase	−1.92	Protein	Metabolic process	mitochondrion;	catalytic activity
16	W5E0G5	Proteasome component	−2.34	Protein	Metabolic process	cytosol	protein binding
17	A0A1D6AAV9	Carbon-nitrogen hydrolase	−2.52	Signaling	Metabolic process	not assigned	catalytic activity
18	A0A1D5TDD8	Plant invertase/pectin methylesterase inhibitor	−2.9	cell wall	Regulation of biological process	membrane	enzyme regulator activity
19	W5E5N6	LysM Domain (Peptidoglycan binding)	−2.9	cell wall	Response to stimulus	membrane	protein binding
20	A0A1D6DBZ9	Polygalacturonase	−3.58	cell wall	Cell organization and biogenesis	extracellular	catalytic activity

Ratio, relative abundance of protein; *p*-value b 0.05. Treated over control, wheat plant treated with Ag NPs compared with control.

## Supplementary Materials

Supplementary materials can be found at http://www.mdpi.com/1422-0067/20/4/825/s1.

## Abbreviations

NPs	Nanoparticles
LC	Liquid Chromatography
ROS	Reactive oxygen species
MS	Mass Spectrometry
SOD	Superoxide dismutase
POD	Peroxidase
CAT	Catalase
