# Supplementary material for: Proteomic Analysis of the Effect of Inorganic and Organic Chemicals on Silver Nanoparticles in Wheat
Source: Int J Mol Sci. 2019 Feb 14;20(4):825. doi: 10.3390/ijms20040825 (PMC6412406; doi:10.3390/ijms20040825)
Supplement: Supplementary file 1 [file ijms-20-00825-s001.pdf]

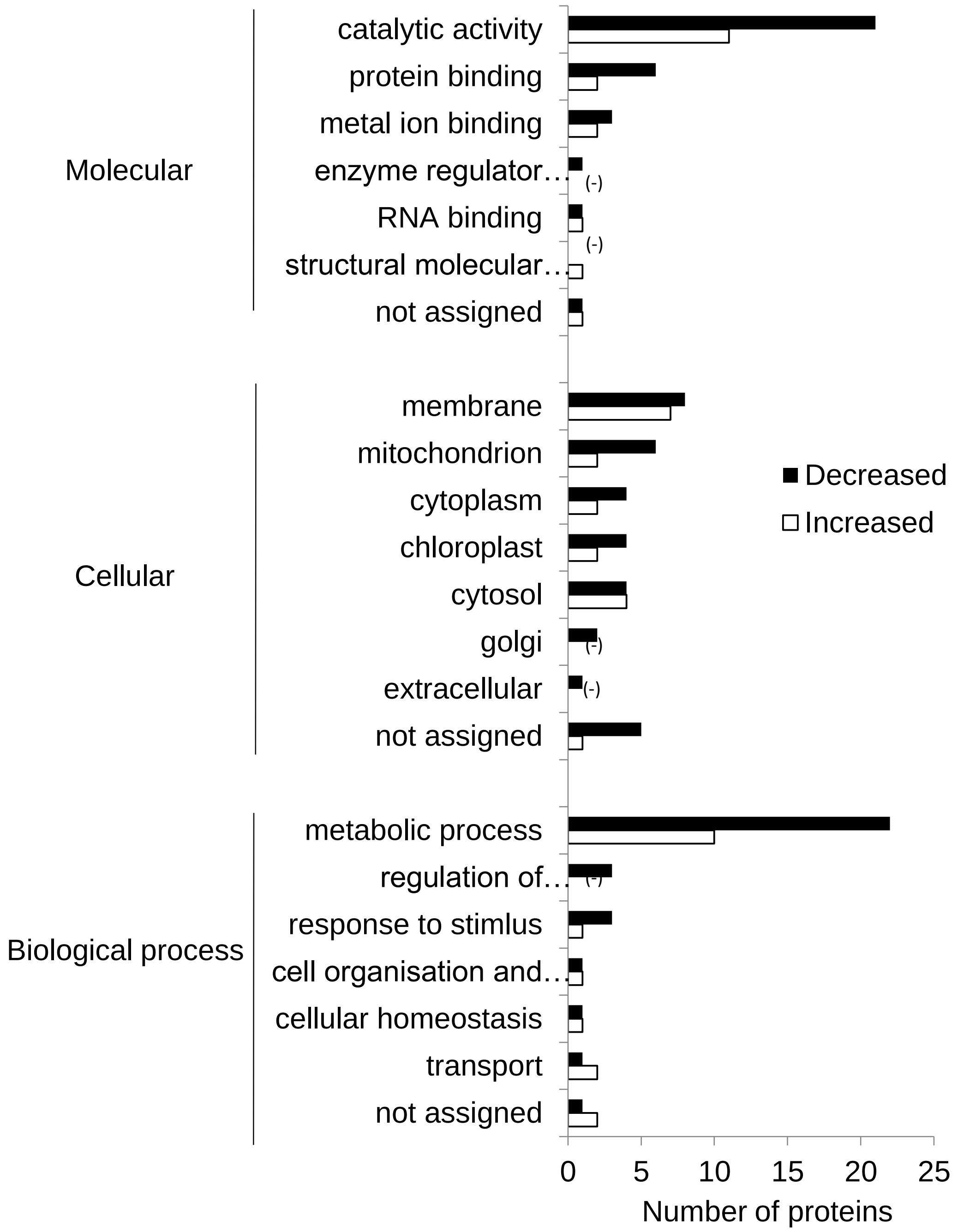

Supplemental Figure 1. GO analysis of identified proteins on chemo-blended Ag NPs exposure. Go categories of proteins with differential abundance in six days old wheat plants treated with Ag NPs blended with organic and inorganic chemicals. Proteins were extracted from shoot and identified through gel free/label free proteomic technique. Proteins with differential abundance were categorized as biological, cellular, and molecular function by WEGO according to Go terms. The x-axis indicated the number of proteins. Categories that contained zero proteins are marked with (-)

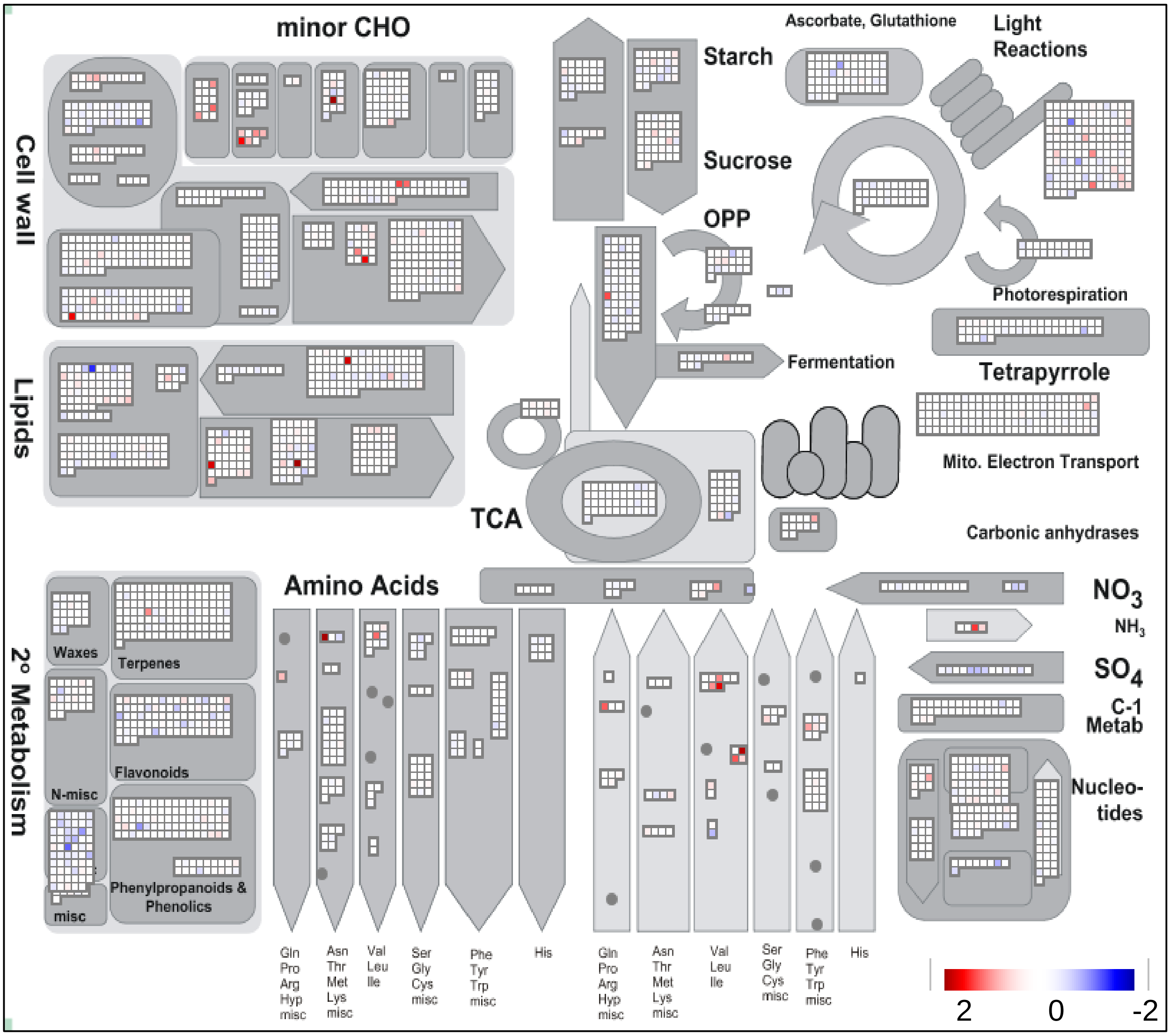

Supplemental Figure 2. MapMan soft analysis of identified proteins on chemo-blended AgNPs exposure. Metabolic pathway of proteins identified in wheat treated with chemo-blended Ag NPs. Wheat plants were treated with Ag NPs mixed with organic and inorganic chemicals for 6 days. The abundance changes of proteins grouped into functional categories related to primary metabolism were visualized using MapMan software. A red and blue color indicates an increase and decrease, respectively, in fold change values compared with untreated one. Abbreviations are as follows; CHO, carbohydrates, TCA,Tricarboxylic acid, OPP, O-propargyl puromycin.
